# Shrinkage estimation of non-negative mean vector with unknown covariance under balance loss

**DOI:** 10.1186/s13660-018-1919-0

**Published:** 2018-12-03

**Authors:** Hamid Karamikabir, Mahmoud Afshari, Mohammad Arashi

**Affiliations:** 1 Department of Statistics, Persian Gulf University, Bushehr, Iran; 2 Department of Statistics, Faculty of Mathematical Sciences, Shahrood University of Technology, Shahrood, Iran

**Keywords:** 62F10, 62J07, Baranchik-type estimator, Balance loss function, Restricted estimator, Shrinkage estimator, Spherical distribution

## Abstract

Parameter estimation in multivariate analysis is important, particularly when parameter space is restricted. Among different methods, the shrinkage estimation is of interest. In this article we consider the problem of estimating the *p*-dimensional mean vector in spherically symmetric models. A dominant class of Baranchik-type shrinkage estimators is developed that outperforms the natural estimator under the balance loss function, when the mean vector is restricted to lie in a non-negative hyperplane. In our study, the components of the diagonal covariance matrix are assumed to be unknown. The performance evaluation of the proposed class of estimators is checked through a simulation study along with a real data analysis.

## Introduction

Shrinkage estimation is a method to improve a raw estimator in some sense, by combining it with other information. Although the shrinkage estimator is biased, it is well known that it has minimum quadratic risk compared to natural estimators (mostly the maximum likelihood estimator).

Mean vector (location) parameter estimation is an important problem in the context of shrinkage estimation, specially when some components of location parameter are restricted to be situated in a specific space. In this respect, Fourdrinier and Ouassou [6] initiated the restricted estimation problem of the mean for the general spherical model with known covariance and Fourdrinier et al. [7] studied the restricted estimation in the latter specified general spherical model, under three different constraints; see also Fourdrinier et al. [9]. Fourdrinier and Marchand [5] studied constraints with the form $\sum_{i=1}^{p} \frac{(\theta_{i} -\tau_{i})^{2}}{\sigma^{2}} \le m^{2} $, with known $\tau_{1}, \dots, \tau_{p}$, $\sigma^{2}$, and *m* when $X_{i} \sim N(\theta_{i}, \sigma^{2})$, $i=1,\dots, p$ on spheres of radius *α* centered at $(\tau_{1}, \dots, \tau_{p})$. Kortbi and Marchand [12] exhibited a truncated linear estimator under the constraint $\Vert\theta\Vert \le m$, in the multivariate normal model. Marchand and Strawderman [16] developed a unified approach for minimax estimation for a restricted parameter space. Kubokawa et al. [13] considered minimax shrinkage estimation of a location vector of a spherically symmetric distribution under a concave squared error loss. Also Chang and Strawderman [3] studied a shrinkage estimation of *p* positive normal means under sum of squared errors loss. Recently, Hoque et al. [10] investigated the performance of the shrinkage estimator of the parameters of a simple linear regression model under the asymmetric loss (LINEX loss criterion). For more details on this topic, we refer to Marchand and Strawderman [15], Silvapulle and Sen [18] and van Eeden [20], among others.

Here, we develop the approach of Fourdrinier et al. [7], in which they estimated location parameter-vector when some components are non-negative, for unknown covariance matrix under balance loss function. We specifically address the Baranchik-type estimators for our purpose.

The paper is outlined as follows: In Sect. 2, some preliminary results are addressed. Section 3 includes the main result, where we give the conditions under which the proposed class of shrinkage estimators dominates the natural estimator under balance loss function, while the numerical performance analysis is investigated by a simulation study in Sect. 4. In Sect. 5, we use the air pollution dataset of USA cities to further demonstrate the superior performance of the shrinkage estimation. The paper is concluded in Sect. 6.

## Preliminaries

In this section, we consider the spherical distribution as the parent model, and introduce the natural and the Baranchik-type shrinkage estimator for estimation of restricted parameter space. A $p\times1$ random vector *X* is said to have a spherically symmetric distribution (or simply spherical distribution) if *X* and *ΛX* have the same distribution for all $p\times p$ orthogonal matrices *Λ*. Important members are the multivariate normal ($N_{p}(0,\sigma^{2} I_{p})$), the “*ε*-contaminated” normal, and multivariate *t* distributions. For evaluating the performance of the estimators, we need to set a measure. In this paper, we use the balance loss function.

### Definition 2.1

Suppose that *X* is a random vector having a spherical distribution with unknown mean vector parameter *θ* and scalar variational component $\sigma^{2}$. the balance error loss function, BEL($\delta_{0}$) is defined as follows:

1 $$ L_{\omega,\delta_{0}}(\theta,\delta) =\omega\frac{ \Vert \delta-\delta_{0} \Vert ^{2}}{\sigma^{2}} + (1- \omega) \frac{ \Vert \delta-\theta \Vert ^{2}}{\sigma^{2}}, \quad0 \le\omega< 1, $$

where $\delta_{0}$ ia a target estimator.

The special case of the balanced error loss function is weighted quadratic loss when $\omega=0$. The balance loss function was introduced by Zellner [21] to reflect two criteria: goodness of fit and precision of estimation. Then the associated risk function with respect to (1), will be $R(\theta, \delta)= E_{\theta} [L(\theta, \delta) ]$. For more details about the use of this loss, we refer to Zinodiny et al. [22], Peng et al. [17], Cao and He [2] and Zinodiny et al. [23], to mention a few.

Assume $(X,U)$ is a $p+k$ random vector having a spherically symmetric distribution around the $p+k$ vector $(\theta,0)$, $\operatorname{dim} X= \operatorname{dim} \theta=p$ and $\operatorname{dim} U= \operatorname{dim} 0=k$. Further, suppose that the scalar variational component $\sigma^{2}$ is unknown which will be posed for *X*. We wish to estimate $\theta= (\theta_{1} , \dots, \theta_{p})^{T}$ by $\delta=(\delta_{1},\dots,\delta_{p})^{T}$ under the balance loss function. Here, we consider the cases where the members of a subset of $\theta_{i} \ge0$, $i=1,\ldots,p$, are non-negative, i.e., $\theta_{1} \ge0 , \theta_{2} \ge0 ,\ldots, \theta_{q} \ge0 $ and where $\theta_{q+1},\theta_{q+2},\ldots ,\theta_{p} $ are unrestricted. Further, let the scale matrix be equal to $\sigma^{2} I_{p}$ with unknown $\sigma^{2}$ and $S^{2}$ is an unbiased estimator of $\sigma^{2}$, independent of *X*.

Define $\gamma_{q}(X)=(\gamma_{q,1}(X),\dots,\gamma_{q,p}(X))$, for $j=1,2, \dots, q$, as

2 $$ \gamma_{q,j}(X)= \textstyle\begin{cases} -X_{j}, &X_{j} < 0,\\ 0, & X_{j}\ge0, \end{cases} $$

and $\gamma_{q,j}(X)=0$ if $j>q$. Then the natural and Baranchik-type shrinkage estimators are, respectively, defined as

3 $$\begin{aligned} &\delta_{q}^{(1)}(X)=X + \gamma_{q}(X), \end{aligned}$$

4 $$\begin{aligned} &\delta_{q}^{(2)}(X,U)=X + \gamma_{q}(X)+ U^{T}U g(X,S), \end{aligned}$$

where $g(X,S)$ has the form

5 $$\begin{aligned} &g(X,S)=-\frac{cS^{2} r(\frac{ \Vert X \Vert ^{2}}{S^{2}})}{ \Vert X \Vert ^{2}}X, \end{aligned}$$

for some constant *c*. Furthermore, suppose that the function $r: \mathbb{R}^{+} \rightarrow[0,1]$ is twice differentiable and concave. To see the original form of the Baranchik-type shrinkage estimators, refer to Baranchik [1]. In the sequel, we need the following results.

### Definition 2.2

A continuous function $f:\mathbb{R}^{p} \rightarrow\mathbb{R}$ is super-harmonic at a point $x_{0} \in\mathbb{R}^{p}$ if, for every $r>0$, the average of *f* over the surface of the sphere $S_{r} (x_{0}) = \{ x : \Vert x-x_{0} \Vert= r \}$ is less than or equal to $f(x_{0})$. The function f is super-harmonic in $\mathbb{R}^{p}$ if it is super-harmonic at each $x_{0} \in\mathbb{R}^{p}$.

### Lemma 2.1

*If* $f:\mathbb{R}^{p} \rightarrow\mathbb{R}$ *is twice differentiable*, *then* *f* *is super*-*harmonic in* $\mathbb{R}^{p}$ *if and only if for all* $x \in\mathbb{R}^{p}$,

6 $$ \nabla\cdot f(x)=\sum_{i=1}^{p} \frac{\partial^{2}}{\partial x_{i}^{2}}f(x) \le0. $$

### Lemma 2.2

*Let* *Y* *be a random variable*, *and* $g(y)$ *and* $h(y)$ *any functions for which* $E[g(Y )]$, $E[(h(Y )]$, *and* $E[g(Y)h(Y)]$ *exist*. *Then*:

- *If one of the functions* $g(\cdot)$ *and* $h(\cdot)$ *is nonincreasing and the other is nondecreasing*,
  $$E\bigl[g(Y )h(Y )\bigr] \le E\bigl[g(Y )\bigr]E\bigl[h(Y )\bigr]. $$
- *If both functions are either nondecreasing or nonincreasing*,
  $$E\bigl[g(Y )h(Y )\bigr] \ge E\bigl[g(Y )\bigr]E\bigl[h(Y )\bigr]. $$

For the proofs of Lemmas 2.1 and 2.2, see Lehmann and Casella [14].

## Main result

In this section, we propose the superiority conditions for which the specified shrinkage estimator (4) outperforms the natural one (3). For our purpose, we consider unimodal spherical distributions. Similar to Jafari Jozani et al. [11], the target estimator can be the part of the shrinkage estimator. Let

7 $$\begin{aligned} &\delta_{0}^{(1)}(X,U)=X + (1-\omega) U^{T}Ug(X,S). \end{aligned}$$

8 $$\begin{aligned} &\delta_{0}^{(2)}(X)=X + (1-\omega) \gamma_{q}(X). \end{aligned}$$

Hence

$$\begin{aligned} &\begin{aligned} \delta_{q}^{(1)}(X)&=\delta_{0}^{(1)}(X,U)+ \gamma_{q}(X)-(1-\omega) U^{T}Ug(X,S) \\ &=\delta_{0}^{(2)}(X)+\omega\gamma_{q}(X), \end{aligned} \\ &\begin{aligned} \delta_{q}^{(2)}(X,U)&=\delta_{0}^{(1)}(X,U)+ \gamma_{q}(X)+\omega U^{T}Ug(X,S) \\ &=\delta_{0}^{(2)}(X)+\omega\gamma_{q}(X)+U^{T}Ug(X,S). \end{aligned} \end{aligned}$$

Considering these two estimators, the difference in risk for $i=1,2$ has the form

9 $$\begin{aligned} \Delta R_{\omega,\delta_{0}^{(i)}}(\theta,\delta) =& R_{\omega,\delta_{0}^{(i)}}\bigl( \theta,\delta_{q}^{(2)} \bigr)-R_{\omega,\delta _{0}^{(i)}}\bigl(\theta, \delta_{q}^{(1)} \bigr) \\ =&\frac{1}{\sigma^{2}}E_{\theta} \bigl[ \omega \bigl( \bigl\Vert \delta_{q}^{(2)} -\delta_{0}^{(i)} \bigr\Vert ^{2} - \bigl\Vert \delta_{q}^{(1)} - \delta_{0}^{(i)} \bigr\Vert ^{2} \bigr) \\ &{}+(1-\omega) \bigl( \bigl\Vert \delta_{q}^{(2)} -\theta \bigr\Vert ^{2} - \bigl\Vert \delta_{q}^{(1)} - \theta \bigr\Vert ^{2} \bigr) \bigr] \\ =&\frac{1}{\sigma^{2}}E_{\theta} \bigl[ \omega \bigl( \bigl\Vert X + \gamma_{q}(X)+ U^{T}U g(X,S) -\delta_{0}^{(i)} \bigr\Vert ^{2} \\ &{} - \bigl\Vert X + \gamma_{q}(X) -\delta_{0}^{(i)} \bigr\Vert ^{2} \bigr) \\ &{}+(1-\omega) \bigl( \bigl\Vert X + \gamma_{q}(X)+ U^{T}U g(X,S) -\theta \bigr\Vert ^{2} \\ &{} - \bigl\Vert X + \gamma_{q}(X) -\theta \bigr\Vert ^{2} \bigr) \bigr] \\ =& \frac{1}{\sigma^{2}} E_{\theta} \bigl[ \bigl(U^{T}U \bigr)^{2} \bigl\Vert g(X,S) \bigr\Vert ^{2} + 2(1-\omega) U^{T}U g^{T}(X,S) (X- \theta) \\ &{}+2(1-\omega) U^{T}U g^{T}(X,S)\gamma_{q}(X) \\ &{} + 2\omega U^{T}U g^{T}(X,S) \bigl( X + \gamma_{q}(X) -\delta_{0}^{(i)} \bigr) \bigr]. \end{aligned}$$

Replacing the estimators $\delta_{0}^{(1)}(X)$ and $\delta_{0}^{(2)}(X)$ in (9), the risk differences for $i=1,2$ are given by the following:

10 $$\begin{aligned} &\begin{aligned}[b] \Delta R^{(1)}={}&\Delta R_{\omega,\delta_{0}^{(1)}}(\theta, \delta) \\ ={}& \frac{1}{\sigma^{2}} E_{\theta} \bigl[ \bigl(U^{T}U \bigr)^{2} \bigl\Vert g(X,S) \bigr\Vert ^{2} + 2(1-\omega) U^{T}U g^{T}(X,S) (X- \theta) \\ &{}+2(1-\omega) U^{T}U g^{T}(X,S)\gamma_{q}(X) \\ &{} + 2\omega U^{T}U g^{T}(X,S) \bigl( \gamma_{q}(X) - (1-\omega) U^{T}Ug(X,S) \bigr) \bigr] \\ ={}& \frac{1}{\sigma^{2}} E_{\theta} \bigl[ \bigl(1-2\omega+2\omega^{2}\bigr) \bigl(U^{T}U \bigr)^{2} \bigl\Vert g(X,S) \bigr\Vert ^{2} \\ &{}+ 2(1- \omega) U^{T}U g^{T}(X,S) (X- \theta) \\ &{}+2U^{T}U g^{T}(X,S)\gamma_{q}(X) \bigr]; \end{aligned} \end{aligned}$$

11 $$\begin{aligned} & \begin{aligned}[b] \Delta R^{(2)}={}&\Delta R_{\omega,\delta_{0}^{(2)}}(\theta, \delta) \\ ={}& \frac{1}{\sigma^{2}} E_{\theta} \bigl[ \bigl(U^{T}U \bigr)^{2} \bigl\Vert g(X,S) \bigr\Vert ^{2} + 2(1-\omega) U^{T}U g^{T}(X,S) (X- \theta) \\ &{}+2(1-\omega) U^{T}U g^{T}(X,S)\gamma_{q}(X) - 2 \omega^{2} U^{T}U g^{T}(X,S)\gamma_{q}(X) \bigr] \\ ={}& \frac{1}{\sigma^{2}} E_{\theta} \bigl[ \bigl(U^{T}U \bigr)^{2} \bigl\Vert g(X,S) \bigr\Vert ^{2} + 2(1-\omega) U^{T}U g^{T}(X,S) (X- \theta) \\ &{}+2\bigl(1-\omega+ \omega^{2}\bigr) U^{T}U g^{T}(X,S) \gamma_{q}(X) \bigr]. \end{aligned} \end{aligned}$$

Inside the expectations (10) and (11), the second term depends on *θ*. To avoid this, we use the following lemmas.

### Lemma 3.1

(Fourdrinier and Strawderman [8])

*For every weakly differentiable function* $g :\mathbb{R} ^{p} \to \mathbb{R}^{p}$, *for every integer* *s* *and for every* $\theta\in \mathbb{R}^{p}$ *we have*

$$ E_{\theta}\bigl[\bigl(U^{T}U\bigr)^{s} g(X,S)^{T}(X-\theta) \bigr]= \frac{1}{k+2s}E_{\theta}\bigl[ \bigl(U^{T}U\bigr)^{s+1} \nabla\cdot g(X,S)\bigr] $$

*provided these expectations exist*.

### Lemma 3.2

(*Stein* [19]) *Suppose that* $X \sim N_{p} (\theta, \sigma^{2} I_{p})$ *and* $g :\mathbb{R} ^{p} \to\mathbb{R}^{p}$ *with known* $\sigma^{2}$, *then*

$$ E_{\theta}\bigl[(X-\theta)^{T} g(X,S)\bigr]= \sigma^{2} E\bigl[\nabla\cdot g(X,S)\bigr]. $$

Taking $s=1$ in Lemma 3.1, for weakly differentiable function *g*, the risk differences (10) and (11) become

12 $$\begin{aligned} &\begin{aligned}[b] \Delta R^{(1)}={}& \frac{1}{\sigma^{2}} E_{\theta} \biggl[ \bigl(1-2\omega+2\omega^{2}\bigr) \bigl(U^{T}U \bigr)^{2} \bigl\Vert g(X,S) \bigr\Vert ^{2} \\ &{}+ \frac{ 2(1-\omega)}{k+2}\bigl(U^{T}U \bigr)^{2} \nabla\cdot g(X,S) \\ &{} +2 U^{T}U g^{T}(X,S)\gamma_{q}(X) \biggr], \end{aligned} \end{aligned}$$

13 $$\begin{aligned} &\begin{aligned}[b] \Delta R^{(2)} ={}& \frac{1}{\sigma^{2}} E_{\theta} \biggl[ \bigl(U^{T}U \bigr)^{2} \bigl\Vert g(X,S) \bigr\Vert ^{2} +\frac{ 2(1-\omega)}{k+2}\bigl(U^{T}U \bigr)^{2} \nabla\cdot g(X,S) \\ &{}+ 2\bigl(1-\omega+\omega^{2}\bigr) U^{T}U g^{T}(X,S)\gamma_{q}(X)\biggr]. \end{aligned} \end{aligned}$$

In order to further analyze the risk difference, we need the following results.

### Lemma 3.3

(Fourdrinier et al. [7])

*If* *r* *is a non*-*negative*, *differentiable and concave real*-*valued function*, *then* *r* *is nondecreasing on* $\mathbb{R}^{+}$ *and the function* $r(t)/t $ *is nonincreasing on* $\mathbb{R}^{+}$. *Furthermore*, *if in addition* *r* *is twice differentiable*, *then the function* $r(\Vert x \Vert^{2})/\Vert x \Vert^{2} $ *is super*-*harmonic for* $p \ge4$.

### Lemma 3.4

*Assume* *X* *is a real*-*valued random variable with symmetric unimodal distribution about* $\theta\in\mathbb{R}^{+}$. *If* *f* *is a non*-*negative function on* $\mathbb{R}^{+} $, *then*

$$ E_{\theta} \biggl[f\bigl(X^{2}\bigr)\frac{X^{2}}{\sigma^{2}} I_{[X< 0]} \biggr] \le \frac{1}{2}E_{\theta} \biggl[ \frac{(X-\theta)^{2}}{\sigma^{2}} f\bigl(X^{2}\bigr) \biggr]. $$

### Proof

According to the symmetry and unimodality of the distribution, *X* has a density of the form $h((X-\theta)^{2})$ with *h* nonincreasing. Thus, we can write

14 $$\begin{aligned} &E_{\theta}\biggl[\frac{f(X^{2})}{\sigma^{2}} \biggl\{ X^{2} I_{[X< 0]}-\frac {1}{2}\bigl(X^{2} -2 \theta X+\theta^{2}\bigr) \biggr\} \biggr] \\ &\quad =E_{\theta}\biggl[\frac{f(X^{2})}{\sigma^{2}} \biggl\{ X^{2} I_{[X< 0]} -\frac{1}{2}\bigl(X^{2} +2 \theta X+ \theta^{2}\bigr)I_{[X< 0]} -\frac{1}{2} \bigl(X^{2} -2 \theta X+\theta^{2}\bigr)I_{[X\ge0]} \biggr\} \biggr] \\ &\quad =E_{\theta}\biggl[ \frac{f(X^{2})}{\sigma^{2}} \biggl\{ \biggl( \frac {1}{2}X^{2} + \theta X - \frac{1}{2} \theta^{2} \biggr) I_{[X< 0]} - \biggl( \frac{1}{2}X^{2} - \theta X + \frac{1}{2}\theta^{2} \biggr) I_{[X\ge0]} \biggr\} \biggr] . \end{aligned}$$

By the conditioning expectation (14) on $|X|$ we have the following expectation:

15 $$\begin{aligned} = &E_{\theta}\biggl[\frac{f(X^{2})}{\sigma^{2}} \biggl( \frac{1}{2}X^{2} + \theta X - \frac{1}{2} \theta^{2} \biggr) I_{[X< 0]}\biggm| \vert X \vert \biggr] \\ &{}- E_{\theta}\biggl[ \frac{f(X^{2})}{\sigma^{2}} \biggl( \frac{1}{2}X^{2} - \theta X + \frac{1}{2}\theta^{2} \biggr) I_{[X\ge0]} \biggm| \vert X \vert \biggr] \\ =& \int_{I_{[\frac{1}{2}X^{2} - \theta \vert x \vert - \frac{1}{2}\theta^{2}>0]}} \frac{f(x^{2})}{\sigma^{2}} \biggl( \frac{1}{2}x^{2} - \theta \vert x \vert - \frac {1}{2}\theta^{2} \biggr) h \bigl(\bigl(- \vert x \vert -\theta\bigr)^{2}\bigr) \,dx \\ &{}- \int_{I_{[\frac{1}{2}x^{2} - \theta \vert x \vert - \frac{1}{2}\theta^{2}>0]}} \frac{f(x^{2})}{\sigma^{2}} \biggl( \frac{1}{2}x^{2} - \theta \vert x \vert + \frac {1}{2}\theta^{2} \biggr) h \bigl(\bigl( \vert x \vert -\theta\bigr)^{2}\bigr) \,dx \\ \le& \int_{I_{[\frac{1}{2}x^{2} - \theta \vert x \vert - \frac{1}{2}\theta ^{2}>0]}} \frac{f(x^{2})}{\sigma^{2}} \bigl(-\theta^{2}\bigr) h\bigl(\bigl( \vert x \vert -\theta\bigr)^{2}\bigr)\,dx \\ =&E_{\theta}\biggl[ \frac{f(x^{2})}{\sigma^{2}} I_{[\frac{1}{2}x^{2} - \theta \vert x \vert - \frac{1}{2}\theta^{2}>0]} \bigl(- \theta^{2}\bigr) \biggr] \le0. \end{aligned}$$

The result follows since in (15), for all $\theta>0$, we have $(-|X|-\theta)^{2}\ge(|X|-\theta)^{2}$ and $h((-|X|-\theta)^{2}) \le h((|X|-\theta)^{2})$. □

We now state the main result.

### Theorem 3.1

*The shrinkage estimator* $\delta_{q}^{(2)}(X,U)$ *dominates the natural estimator* $\delta_{q}^{(1)}(X)$ *under the BEL*($\delta_{0}^{(1)}$), *if the following conditions hold*:

1. $p>\frac{q(k+2)}{2 (1-\omega)(k-2)}+2$,
2. $0 < c \le\frac{ (2 (1-\omega)\frac{p-2}{k+2} - \frac{q}{k-2} )}{(1-2\omega+2\omega^{2}) }\frac{E_{\sigma =1}(S^{2})}{E_{\sigma=1}(S^{4})}$.

### Proof

Since $0\le r(\cdot) \le1$ is a non-negative, differentiable and concave function by Lemma 3.3, we have $r^{\prime}(\cdot)\ge0$. Using Lemma 3.1 and by the conditioning risk difference $\Delta R^{(1)}$ on $(S=s)$, we have the following inequality:

16 $$\begin{aligned} &\frac{1}{\sigma^{2}}E_{S^{2}} \Biggl( E_{\theta} \Biggl[ c^{2}\bigl(1-2\omega+2\omega^{2}\bigr) \bigl(U^{T}U \bigr)^{2} \frac {r^{2}(\frac{ \Vert X \Vert ^{2}}{S^{2}})S^{4}}{ \Vert X \Vert ^{2}} \\ &\qquad {} -4c(1-\omega)\frac{ (U^{T}U )^{2} r^{\prime}(\frac{ \Vert X \Vert ^{2}}{S^{2}})}{k+2} \\ &\qquad {} -2c(1-\omega)\frac{(p-2) (U^{T}U )^{2}r(\frac{ \Vert X \Vert ^{2}}{S^{2}})S^{2}}{(k+2) \Vert X \Vert ^{2}} \\ &\qquad {}+2c \bigl(U^{T}U\bigr)\frac{ r(\frac{ \Vert X \Vert ^{2}}{S^{2}})S^{2}}{ \Vert X \Vert ^{2}}\sum _{i=1}^{q} X_{i}^{2} I_{[X_{i} \le0]} \Biggr] \Biggm|S=s \Biggr) \\ &\quad \le \frac{1}{\sigma^{2}}E_{S^{2}} \biggl( E_{\theta} \biggl[ \bigl(U^{T}U \bigr)^{2} \frac{r(\frac{ \Vert X \Vert ^{2}}{S^{2}})}{ \Vert X \Vert ^{2}} c \biggl( c \bigl(1-2\omega+2\omega^{2}\bigr) S^{4} -2(1-\omega) S^{2}\frac {p-2}{k+2} \\ &\qquad {}+2 S^{2} \frac{\sum_{i=1}^{q} X_{i}^{2} I_{[X_{i} \le0]}}{U^{T}U} \biggr) \biggr]\biggm|S=s \biggr). \end{aligned}$$

Suppose $X_{1}^{q}=(X_{1},\ldots,X_{q})$, $\eta=(\theta_{1},\ldots,\theta_{q})$, $X_{q+1}^{p}=(X_{q+1},\ldots,X_{p})$, $\mu=(\theta_{q+1},\ldots,\theta_{p})$, $Z=\sigma^{-1}(X-\theta)$, $V=(Z_{1},\ldots,Z_{q})$ and $T=(Z_{q+1},\ldots ,Z_{p})$. Hence, $V=\sigma^{-1}(X_{1}^{q}-\eta) $ and $T=\sigma ^{-1}(X_{q+1}^{p}-\mu)$. Since $\Vert X\Vert^{2}=\Vert X_{1}^{q} \Vert^{2}+\Vert X_{q+1}^{p}\Vert^{2}$, $X_{1}^{q}=\sigma V+\eta$ and $X_{q+1}^{p}=\sigma T+\mu$. Let $W^{2}=V'V + U'U$. Then, assuming $\sigma=1$, an upper bound on the conditional expression (16) by Lemma 3.4 is given by

17 $$\begin{aligned} & E_{S^{2}} \biggl\{ E_{\theta} \biggl[ \bigl(W^{2}-V^{T}V\bigr)^{2} \frac{r(( \Vert X_{1}^{q} \Vert ^{2} + \Vert X_{q+1}^{p} \Vert ^{2} )/S^{2})}{ \Vert X_{1}^{q} \Vert ^{2} + \Vert X_{q+1}^{p} \Vert ^{2}} c \biggl( c\bigl(1-2\omega+2\omega^{2}\bigr)S^{4} \\ &\qquad{}-2(1-\omega)S^{2}\frac{p-2}{k+2}+ S^{2} \frac{V^{T} V}{W^{2}-V^{T}V} \biggr) \biggr] \biggm|S=s \biggr\} . \end{aligned}$$

Using Lemma 3.3, $\frac{r ( (\Vert X_{1}^{q}\Vert^{2} +\Vert X_{q+1}^{p}\Vert^{2} ) /S^{2} )}{\Vert X_{1}^{q}\Vert^{2} +\Vert X_{q+1}^{p}\Vert^{2}}$ for $p\ge4$ is super-harmonic and as a result, in $\frac{\Vert X\Vert ^{2}}{S^{2}}$, is nondecreasing. Therefore, the conditional risk difference (17) given $W^{2}$ and *T* is

18 $$\begin{aligned} &cE_{\theta} \biggl[\bigl(W^{2}-V^{T}V \bigr)^{2} \frac{r(( \Vert X_{1}^{q} \Vert ^{2} + \Vert X_{q+1}^{p} \Vert ^{2} )/s^{2})}{ \Vert X_{1}^{q} \Vert ^{2} + \Vert X_{q+1}^{p} \Vert ^{2}} \\ &\qquad {}\times \biggl( c\bigl(1-2\omega+2\omega^{2}\bigr)s^{4} -2(1-\omega)s^{2}\frac {p-2}{k+2}+ s^{2} \frac{V^{T} V}{W^{2}-V^{T}V} \biggr)\biggm| W^{2},T \biggr] \\ &\quad \le c E_{\theta} \biggl[\bigl(W^{2}-V^{T}V \bigr)^{2} \frac{r(( \Vert X_{1}^{q} \Vert ^{2} + \Vert X_{q+1}^{p} \Vert ^{2} )/s^{2})}{ \Vert X_{1}^{q} \Vert ^{2} + \Vert X_{q+1}^{p} \Vert ^{2}} \biggm| W^{2},T \biggr] \\ &\qquad {}\times E_{\theta} \biggl[ \biggl( c\bigl(1-2\omega+2\omega^{2} \bigr)s^{4} -2(1-\omega )s^{2}\frac{p-2}{k+2} \\ &\qquad{}+ s^{2} \frac{V^{T} V}{W^{2}-V^{T}V} \biggr)\biggm| W^{2},T \biggr]. \end{aligned}$$

In equality (18), by Lemma 2.2, for fixed $W^{2}$ and *T*, we see that $E_{\theta} [ \frac{r ((\Vert X_{1}^{q}\Vert^{2} +\Vert X_{q+1}^{p}\Vert^{2} )/s^{2} )}{\Vert X_{1}^{q}\Vert^{2} +\Vert X_{q+1}^{p}\Vert^{2}} | W^{2},T ]$ is nonincreasing in $V^{T}V $ by Lemma A.4 of Fourdrinier et al. [7]. It suffices to show that the second conditional expectation in (18) is non-positive. Since $U^{T}U$ and $V^{T} V$ have distributions $\chi_{k}^{2}$ and $\chi_{q}^{2}$, respectively, $(V^{T}V)/W^{2}$ is distributed according to $Beta(\frac{q}{2},\frac{k}{2})$ and hence we get

$$ E\bigl[V^{T}V/\bigl(W^{2}-V^{T}V\bigr) \bigr]=q/(k-2). $$

Then the risk difference is non-positive if

$$ 0 < c \le\frac{ (2 (1-\omega)\frac{p-2}{k+2} - \frac{q}{k-2} )}{(1-2\omega+2\omega^{2}) }\frac{E_{\sigma =1}(S^{2})}{E_{\sigma=1}(S^{4})}. $$

Simple calculations show that *c* is positive if and only if

$$ k > \frac{4(1-\omega)(p-2)+2q }{2(1-\omega)(p-2) -q}. $$

This completes the proof. □

In a similar fashion, we have the following result, stated without proof.

### Theorem 3.2

*The shrinkage estimator* $\delta_{q}^{(2)}(X,U)$ *dominates the natural estimator* $\delta_{q}^{(1)}(X)$ *under the BEL*($\delta_{0}^{(2)}$), *if the following conditions hold*:

1. $p >\frac{q(1-\omega+\omega^{2})(k+2)}{2 (1-\omega)(k-2)}+2$,
2. $0 < c \le (2 (1-\omega)\frac{p-2}{k+2} - (1-\omega+\omega^{2})\frac{q}{k-2} ) \frac{E_{\sigma =1}(S^{2})}{E_{\sigma=1}(S^{4})}$.

The following result is for the *p*-variate normal distribution, a particular member of the spherical class.

### Proposition 3.1

*Assume the parent distribution* $N_{p} (\theta, \sigma^{2} I_{p})$ *with unknown* $\sigma^{2}$. *Then the shrinkage estimator* $X + \gamma_{q}(X)+ g(X,S)$ *dominates the natural estimator* $X + \gamma_{q}(X)$ *under the BEL*($\delta_{0}^{(i)}$), *if the following conditions hold*:

1. *For BEL*($\delta_{0}^{(1)}$): $p> \frac{q}{2(1-\omega) }+2$, $0 < c \le\frac{ (2 (1-\omega)(p-2) -q )}{(1-2\omega+2\omega ^{2}) }\frac{E_{\sigma=1}(S^{2})}{E_{\sigma=1}(S^{4})}$.
2. *For BEL*($\delta_{0}^{(2)}$): $p> \frac{(1-\omega+\omega^{2})q}{2(1-\omega) }+2$, $0 < c \le (2 (1-\omega)(p-2) - (1-\omega+\omega^{2})q ) \frac{E_{\sigma=1}(S^{2})}{E_{\sigma=1}(S^{4})}$.

### Proof

The proof is similar to that of Theorem 3.1. However, we use Lemma 3.2, instead of Lemma 3.1. □

## Simulation

**Figure 1:**
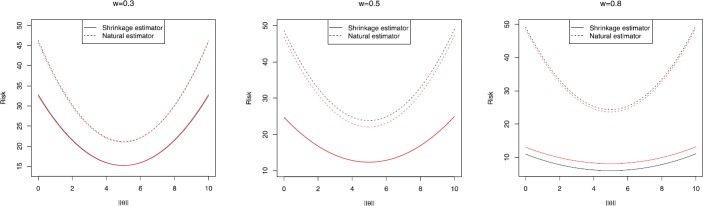
Risk curve for $\delta_{0}^{(1)}(X)$, $p=14$, black line for $q=5$ and red line for $q=10$ for different values of *ω*

**Figure 2:**
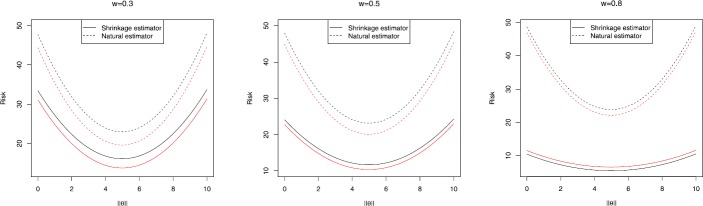
Risk curve for $\delta_{0}^{(2)}(X)$, $p=14$, black line for $q=5$ and red line for $q=10$ for different values of *ω*

To evaluate the performance of a Baranchik-type shrinkage estimator, in this section, we conduct a Monte Carlo simulation study to compare its risk with that of the natural estimator for the 14-variate *t* distribution with 13 degrees of freedom. Risk values are obtained from 1000 Monte Carlo replications, and plotted in Figs. 1 and 2, for different values *q* and *w*. In these figures *θ* is selected as $( {j,0, \ldots,0} )$ and $j=0,0.1,0.2,\ldots , 10$. In this case, $\| \theta\| = {\theta^{T}}\theta = \sum_{i = 1}^{p} {{\theta_{i}}} = {j^{2}}$.

In Figs. 1 and 2, the (Baranchik-type) shrinkage estimator risk curve is below that of the natural estimator, i.e., the shrinkage estimator dominates the natural estimator. Further, it is seen by increasing the amount of *w*, the risk difference gets larger, which is a bonus in our study.

## Air pollution data

In this section, we further investigate the superior performance of the Baranchik-type shrinkage estimator compared to the natural estimator. For this sake, we use the air pollution dataset of USA cities in 1981, from Everitt and Hothorn [4]. They fitted a *p*-variate normal distribution to this dataset. Here, we have the following list of variables: SO2 content of air in micrograms per cubic meter (*SO2*), average annual temperature in degrees Fahrenheit (*temp*), number of manufacturing enterprises employing 20 or more workers (*manu*), population size (1970 census) in thousands (*popul*), average annual wind speed in miles per hour (*wind*), average annual precipitation in inches (*precip*), average number of days with precipitation per year (*predays*). We have implemented a bootstrap analysis to evaluate the risk functions. Table 1 lists the values of risk difference $(\Delta R^{(i)})$ for different values of *w* and $\sigma^{2}$, for targeted estimators $\delta_{0}^{(1)}(X)$ and $\delta_{0}^{(2)}(X)$, respectively. All the values in these tables are negative. (A negative value is a sign of $R_{\omega,\delta_{0}^{(i)}}(\theta,\delta_{q}^{(2)} ) \le R_{\omega ,\delta_{0}^{(i)}}(\theta, \delta_{q}^{(1)} )$.) The same conclusions as for the figures in the previous section can also be obtained.

**Table 1:** Values of risk difference for $p=7$

(*ω*,*q*)	(0.3,5)	(0.5,3)	(0.7,2)
$\Delta R^{(1)}$	−0.0004641284	−0.0003845636	−0.0000994561
$\Delta R^{(2)}$	−0.0004105216	−0.0002643874	−0.0000819120

## Conclusion

In this paper, the estimation of a restricted parameter space is considered using a class of general shrinkage type estimators under a balance loss function. The class of Baranchik-type shrinkage estimators is considered as a competitor to the well-known James–Stein ones. Since the scalar scale component was unknown, we used another random variable, $S^{2}$ say, independent from the model under study. Theoretical findings of this paper are further supported by some numerical analyses. It is observed that the Baranchik-type shrinkage estimator is always superior to the natural estimator, regardless of the weight value in balance loss function. The result of this paper can stimulate the research in the direction of the mean estimation in restricted parameter space.
